# Antitumor enhancement of celecoxib, a selective Cyclooxygenase-2 inhibitor, in a Lewis lung carcinoma expressing Cyclooxygenase-2

**DOI:** 10.1186/1756-9966-27-66

**Published:** 2008-11-11

**Authors:** Won Park, Young Taek Oh, Jae Ho Han, Hongryull Pyo

**Affiliations:** 1 Department of Radiation Oncology, Samsung Medical Center, Sungkyunkwan University School of Medicine, Seoul, Korea; 2 Department of Radiation Oncology, Ajou University Hospital, Suwon, Korea; 3 Department of Pathology, Ajou University Hospital, Suwon, Korea; 4 Research Institute and Hospital, National Cancer Center, Goyang, Korea

## Abstract

**Background:**

The goal of this study was to determine the effects of a selective Cyclooxygenase (COX)-2 inhibitor on the inhibition of tumor growth and pulmonary metastasis in a Lewis Lung Carcinoma (LLC) animal model.

**Methods:**

For immunoblot analysis of COX-2 and PGE2, cells were treated with irradiation in the presence or absence of celecoxib. The right thighs of male, 6-week old C57/BL mice were subcutaneously injected with 1 × 10^6 ^LLC cells. The animals were randomized into one of six groups: (1) no treatment, (2) 25 mg/kg celecoxib daily, (3) 75 mg/kg celecoxib daily, (4) 10 Gy irradiation, (5) 10 Gy irradiation plus 25 mg/kg celecoxib daily, and (6) 10 Gy irradiation plus 75 mg/kg celecoxib daily. Mice were irradiated only once, and celecoxib was administered orally. Mice were irradiated with 4-MV photons once the tumor volume of the control group reached 500 mm^3^. All mice were sacrificed when the mean tumor volume of control animals grew to 4000 mm^3^. The left lobes of the lungs were extracted for the measurement of metastatic nodules.

**Results:**

Irradiation resulted in a dose-dependent increase in PGE2 production. PGE2 synthesis decreased markedly after treatment with celecoxib alone or in combination with irradiation. Compared to mice treated with low dose celecoxib, mean tumor volume decreased significantly in mice treated with a high dose of celecoxib with or without irradiation. Mice treated with a high dose celecoxib alone, with irradiation alone, or with irradiation plus celecoxib had markedly fewer metastatic lung nodules than controls. The mean metastatic area was the smallest for mice treated with irradiation plus a high dose celecoxib.

**Conclusion:**

Oral administration of high dose celecoxib significantly inhibited tumor growth, as compared to a low dose treatment. Radiotherapy in combination with high dose celecoxib delayed tumor growth and reduced the number of pulmonary metastases to a greater extent than celecoxib or radiotherapy alone.

## Background

Radiotherapy is a common treatment for localized cancers. The radiation dose is important for tumor control. However, the therapeutic efficacy of radiotherapy is often limited by normal tissue damage within or nearby the field of radiation. In clinical practice, the radiation dose is optimized according to the probability of tumor control compared to the risks of complications due to the effects on normal tissue [1,2]. Combining chemotherapeutic agents concurrently with radiotherapy has improved tumor control and survival. However, this combined approach also increases systemic and local toxicities during radiotherapy. Because of the increased toxicity, the overall treatment duration of radiotherapy, in addition to chemotherapy, is usually prolonged when compared to the treatment time of radiotherapy alone [3,4]. This increased duration may decrease its efficacy for tumor control within the radiation field.

To further improve tumor response and reduce normal tissue toxicity from radiotherapy or chemoradiotherapy, many novel approaches have investigated several agents in preclinical and clinical settings. These approaches include those that selectively interfere with certain molecular processes and signaling pathways that regulate proliferation, survival, and function of normal cells. Because these agents are preferentially associated with specific sites of the cancer cells, their targeting is predicted to improve the tumor response to radiotherapy or chemoradiotherapy without additional toxicity to normal tissue. Among these agents, inhibition of cyclooxygenase (COX)-2 has been investigated as a potentially useful agent for the treatment of cancer.

COX-2 is normally present in cells and tissues of the brain and kidneys, but is induced in pathological states such as inflammation and tumors. COX-2 promotes carcinogenesis, tumor proliferation, angiogenesis, prevention of apoptosis, and immunosuppression [5]. COX-2 overexpression has been associated with tumor behavior and prognosis in several cancers [6]. Selective inhibition of COX-2 activity in several animal models has been associated with the decrease of new blood vessel production in tumors, a decrease in new vessel formation and an increase in tumor cell apoptosis. The selective inhibition of COX-2 activity has been associated with enhanced radiation sensitivity of tumors without enhancing the effects of radiation on normal tissue [7-9].

In this study, we evaluated the effect of a selective COX-2 inhibitor as a radiation sensitizer in order to inhibit tumor growth and pulmonary metastasis in a Lewis Lung Carcinoma (LLC) animal model.

## Methods

### Animals and Tumor Cells

Male, 6-week old C57/BL mice (Ajou animal laboratory, Suwon, Korea) were used for these experiments. The mice were acclimated for 1 week, and caged in groups of five or less in an air conditioned room. Mice were fed a diet of animal chow and water *ad libitum*. LLC cells were purchased from the American Type Tissue Collection. LCC cells were maintained in DMEM supplemented with 10% fetal bovine serum and penicillin-streptomycin. Cells were grown in monolayers in 100 mm dishes, and were maintained in a humidified 5% CO2 incubator at 37°C.

### Celecoxib

Stock solutions of celecoxib were made by dissolving the compound in DMSO, then were stored at -20°C. Concentrated drug stocks were diluted in DMEM before administration to cells or mice.

### Immunoblot Analysis of COX-2

Cells were pretreated with 10 or 30 μM celecoxib for 1 h at 37°C. After treatment, the cells were irradiated at a dose of 5 Gy or 10 Gy. At 24 or 48 h post treatment, the cells were washed twice with PBS and lysed in buffer (Upstate). Supernatant protein concentrations were determined by Bradford assay using bovine serum albumin (BSA, Sigma Chemical Co.) as a standard. Aliquots of total protein (40 μg) was denatured and fractionated by SDS-polyacrylamide gel electrophoresis (4–12% gels). The separated proteins were transferred to a 0.22 μm nitrocellulose membrane. The nonspecific binding sites were blocked for 1 h in 5% non-fat dry milk and in Tris-buffered saline (TBS). The membranes were incubated with monoclonal anti-COX-2 (610204, BD biosciences) and anti-α tubulin (Oncogene) for approximately 1 h at room temperature. The membranes were washed in buffer containing TBS plus 0.05% Tween-20 and incubated in the appropriate secondary antibody (P0447, Dakocytonation). Signals were detected using enhanced chemiluminescence (Pierce).

### Determination of PGE2 Synthesis

1 × 10^6 ^cells were either untreated, or treated with 30 μM celecoxib for 1 hr and then with 0, 5, or 10 Gy irradiation. After each treatment, supernatant PGE2 levels were assayed in triplicate. Determination of PGE2 levels by enzyme immunoassay was accomplished using a PGE2 monoclonal enzyme immunoassay kit (Cayman Chemical). Quantification was performed according to the manufacturer's instructions.

### In vivo Tumor Growth and Quantitation of Lung Metastases

A suspension of 1 × 10^6 ^LLC cells in 0.1 ml of growth medium was injected subcutaneously into the shaved right thighs of mice. The study groups (n = 12 per group) consisted of an untreated control (group 1), 25 mg/kg celecoxib daily (group 2), 75 mg/kg celecoxib daily (group 3), 10 Gy irradiation (group 3), 10 Gy irradiation plus 25 mg/kg celecoxib daily (group 5), and 75 mg/kg 10 Gy irradiation plus celecoxib daily (group 6).

Celecoxib was administered by lavage (0.1 mL) every afternoon from one day before the cell injection until the day of euthanasia or death. For tumor irradiation, mice were put under general anesthesia and restrained using adhesive tape and customized devices constructed from a 50 ml syringe. Once the tumors in the control group reached a mean volume of 500 mm^3^, the tumors in the right thighs were irradiated with 10 Gy using a 4 MV x-ray for one fraction. Following injection of the tumor cells, the primary tumors were measured three times a week at two perpendicular diameters using a Vernier caliper, and tumor volumes were evaluated based on the formula, volume = 0.5 × a × b^2 ^where a = length and b = width.

All mice were euthanized when the mean tumor volume in the control group reached 4000 mm^3^. The left lobes of the lungs were extracted, fixed in 10% formalin, and processed for the quantitation of metastatic nodules. The number of metastatic nodules was measured in the maximum sagittal plane from 5 μm paraffin-embedded lung tissue sections. The dimension of the outlined metastatic nodule was automatically calculated using commercial software (*i *solution DT, Seoul, Korea).

### Statistical Analysis

The primary tumor volumes were expressed as the mean and standard deviation. Comparison of the area of the lung nodules and tumor volumes among the experimental groups was determined by the Wilcoxon rank-sum test. The correlation between tumor volume and area of the metastatic lung nodules was evaluated by regression analysis. *P-*values less than 0.05 were considered statistically significant. All statistical analyses were performed with the SAS^® ^System (SAS 14.0, SAS Institute Inc., Cary, NC., USA).

## Results

### In vitro, Effect of the Selective COX-2 Inhibitor on LLC Cells

**Figure 1:**
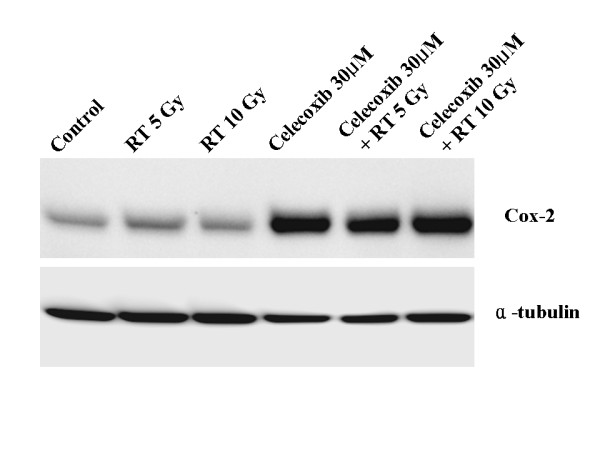
**Celecoxib effects on COX-2 protein expression.** Western blot analysis showed constitutive expression of COX-2 protein in vehicle treatment as a control.

**Figure 2:**
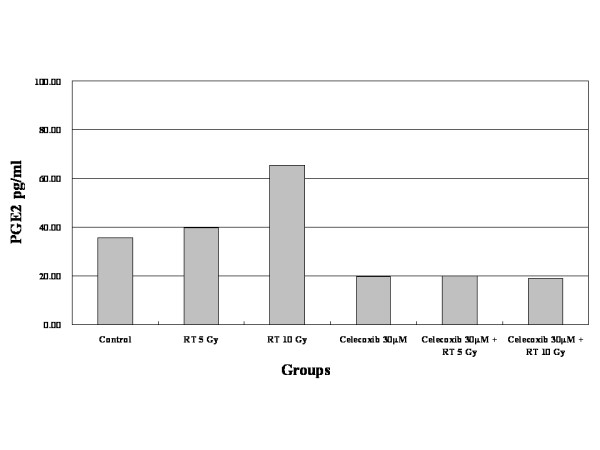
**Celecoxib effects on PGE2 production. **PGE2 production (normalized to 10^6^ cells) by ELISA in Lewis Lung Carcinoma cells after irradiation with single doses of 5 and 10 Gy alone and in combination with 30 μM.

LLC cell COX-2 protein expression was confirmed by western blot analysis, which showed constitutive COX-2 expression (Figure 1). Differences in the amount of COX-2 protein expression were not observed after irradiation alone. However, COX-2 expression increased with celecoxib treatment alone and with irradiation plus celecoxib. Irradiation was associated with a dose-dependent increase in PGE2 production, as measured by enzyme immunoassay (Figure 2). PGE2 synthesis decreased markedly after treatment with celecoxib alone or with celecoxib in combination with irradiation. Radiation treatment plus celecoxib did not increase PGE2 production when compared to celecoxib alone, regardless of the radiation dose.

### In vivo Tumor Growth

**Figure 3:**
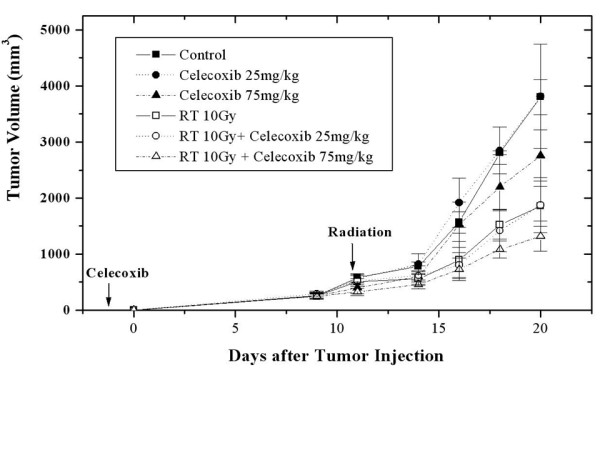
**Celecoxib effects the in vivo tumor growth of Lewis lung carcinoma cells.** Dose dependent delay of tumor growth with celecoxib in mice regardless of irradiation.

When the mean volume of the tumors in the control group reached 539.6 mm^3 ^(487~589 mm^3^), 11 days after LLC cell injection, the animals in irradiation alone or celocoxib plus irraditation were irradiated. At 20 days after injections, a protruding tumor and areas of denuded surface were found at the injection sites, especially in control mice. The mean tumor volume in the control group increased to 3802.2 mm^3 ^(3466~4332 mm^3^), and all mice were euthanized as planned. At that time, the mean tumor volumes of groups 2, 3, 4, 5, and 6 were 3814.4 mm^3 ^(2225~5421 mm^3^), 2757.6 mm^3 ^(1995~3395 mm^3^), 1942.1 mm^3 ^(1766~2275 mm^3^), 1874.3 mm^3 ^(1341~2437 mm^3^), and 1319.3 mm^3 ^(989~1815 mm^3^), respectively (Figure 3).

Compared to the control group, the mean tumor volume decreased significantly in all treatment groups, except for those treated with 25 mg/kg celecoxib daily (Table 1). The effect of celecoxib on the delay of tumor growth was dose-dependent. Compared to the mice treated daily with 25 mg/kg celecoxib, the mean tumor volumes decreased significantly in the mice treated daily with 75 mg/kg celecoxib, with or without irradiation (p = 0.0135, p = 0.0144). The delay in tumor growth was evident in the irradiated groups, regardless of the celecoxib dose.

**Table 1:** Wilcoxon rank-sum test for mean tumor volume at 20 days after tumor cell injection in control and treatment groups (p-value).

Groups	Control	Celecoxib 25 mg/kg	Celecoxib 75 mg/kg	RT	RT + Celecoxib 25 mg/kg	RT + Celecoxib 75 mg/kg
Control	-	0.9761	0.0004	< 0.0001	< 0.0001	< 0.0001

Celecoxib 25 mg/kg	0.9761	-	0.0135	< 0.0001	0.0002	< 0.0001

Celecoxib 75 mg/kg	0.0004	0.0135	-	0.0004	0.0023	< 0.0001

RT	< 0.0001	< 0.0001	0.0004	-	0.7238	0.0001

RT + Celecoxib 25 mg/kg	< 0.0001	0.0002	0.0023	0.7238	-	0.0144

RT + Celecoxib 75 mg/kg	< 0.0001	< 0.0001	< 0.0001	0.0001	0.0144	-

RT: radiation

### In vivo Lung Metastasis

**Figure 4:**
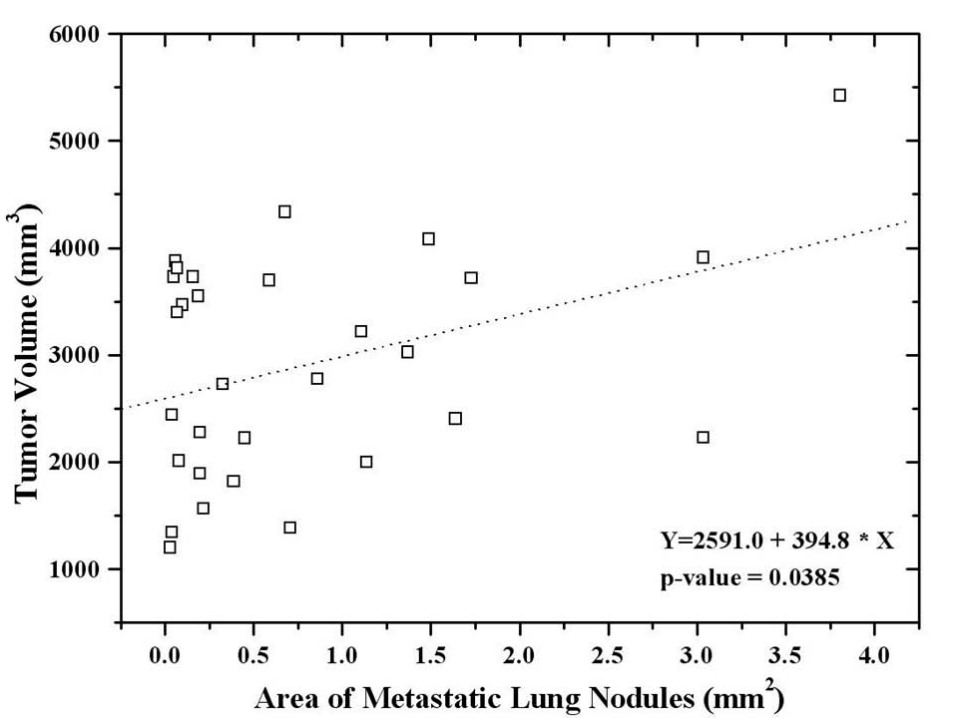
Regression analysis showed a significant correlation between the area of metastatic lung nodules and tumor volume regardless of treatment.

The prevalence of detected lung metastases from groups 1 to 6 were 100%, 100%, 75.0%, 50.0%, 87.5%, and 25%, respectively. The mean area of the metastatic lung nodules did not differ between mice treated with low dose celecoxib (25 mg/kg) only and the control group (Table 2). However, the area of metastatic lung nodules decreased significantly in mice treated with high dose celecoxib (75 mg/kg) alone or with irradiation with or without celecoxib. Among the mice treated with irradiation, the mean metastatic area was smaller than in the control group. The mean metastatic area was the smallest in mice treated with irradiation and 75 mg/kg celecoxib daily (group 6); however, the difference was only marginally significant (p = 0.0675). The area of metastatic lung nodules significantly correlated with tumor volume, regardless of treatment (Figure 4).

**Table 2:** Analysis of metastatic lung nodules according to control and treatment groups.

Groups	Areas of metastatic nodule (mean, mm^2^)	p-value
Control	0.61–4.04 (1.09)	-

Celecoxib 25 mg/kg	0.05–3.81 (1.48)	0.3607

Celecoxib 75 mg/kg	0.07–1.37 (0.81)	0.0295

RT	0.20–0.45 (0.31)	0.0181

RT + Celecoxib 25 mg/kg	0.04–1.64 (0.50)	0.0238

RT + Celecoxib 75 mg/kg	0.03–0.22 (0.13)	0.0675

RT: radiation

## Discussion

COX-2 expression has been reported in a variety of human cancers such as breast, lung, colon, cervix, liver, and prostate. Among tumors types, the intensity or distribution of COX-2 varies. However, COX-2 overexpression has been associated with tumor differentiation, tumor size, stage, and metastasis. In several cancers, patients with COX-2 overexpression have a poor prognosis [10-14]. Denkert et al. immunohistochemically evaluated COX-2 expression from breast cancer specimens. They reported more lymph node metastasis, larger tumors, poor differentiation, increased vascular invasion, and negative estrogen receptor status in patients with elevated COX-2 expression. COX-2 overexpression was reported to be of borderline significance for disease-free survival (relative risk 1.90). Sheehan et al. showed that high COX-2 expression correlates with advanced stage disease and larger tumors in patients with colorectal cancer.

COX-2 associates with tumor growth, infiltration, and metastasis in preclinical experiments. Tsujii et al. studied the phenotypic and biochemical changes associated with COX-2 expression. Rat intestinal epithelial cells were infected with a COX-2 expression vector oriented in the sense (RIE-S) or anti-sense (RIE-AS) direction. RIE-S cells demonstrated increased adhesion to extracellular matrix proteins and inhibition of apoptosis, compared with RIE-AS cells. After human colon cancer cells (Caco-2) were transfected with a COX-2 expression vector, Caco-2 cells acquired increased invasiveness compared with parental cells [15,16]. Liu et al. showed that transgenic mice overexpressing COX-2 in the mammary gland, expressed COX-2 during pregnancy and lactation. Multiparous transgenic mice, but not virgin mice, exhibited a high incidence of focal mammary gland hyperplasia, dysplasia, and transformation into metastatic tumors [17].

Nonsteroidal anti-inflammatory drugs (NSAID) are widely used for relief of inflammatory pain worldwide. Several population-based reports have demonstrated a 40% decrease in the death rate in persons with digestive tract cancers who regularly used aspirin, compared to those who did not. Clinical trials with NSAID in patients with Familial Adenomatous Polyposis clearly demonstrated that NSAID treatment shrunk pre-existing adenomas [18-20]. In addition, indomethacin offered palliative support to patients with advanced solid tumors, and prolonged their mean survival compared to placebo-treated patients [21]. The mechanism of NSAID action was unknown until in 1971, when Vane proposed that NSAIDs primarily suppress inflammation by inhibiting COX and by limiting the production of PG [22]. NSAIDs non-selectively inhibit the activities of both COX-1 and COX-2. COX-1 inhibition causes the adverse effects of NSAIDs on the gastrointestinal tract [23,24]. In a randomized, placebo-controlled endoscopic evaluation, Scheiman et al. reported that the short-term use of nonselective COX inhibitors associated with a significantly greater ulceration rate, compared with a placebo [25].

The development of selective COX-2 inhibitors, with decreased potential for gastrointestinal toxicity, has stimulated additional investigations. Numerous studies on the antineoplastic effects of selective COX-2 inhibitors have been performed. Williams et al. proposed that celecoxib reduced the viability of cell lines, including LLC cells, from the induction of apoptosis and the growth of tumors *in vivo*, and had no effect on apoptosis or the epithelium of the normal gut [26]. Leahy et al. reported that a reduction in proliferation and an increase in apoptosis were observed in colorectal tumor cells in response to celecoxib [27]. Connolly et al. proposed that *in vitro *COX inhibitors decreased vascular endothelial growth factor production and increased apoptosis of tumor cells, as well as a reduced primary tumor weight, the number of lung metastases, and microvessel density in primary tumors in mice [28]. Kobayashi et al. showed that selective COX-2 inhibition in colon cancer cell lines reduced the diameter of the tumor vessels as well as the number and size of the metastatic nodules in the lung. In addition, dose-dependent selective COX-2 inhibition reduced the size of metastatic tumors [9].

In our study, we used western blot analysis to confirm that a COX-2 inhibition increased COX-2 expression. The effect of celecoxib on the delay of tumor growth was dose-dependent, regardless of irradiation. High dose celecoxib (75 mg/kg celecoxib daily) significantly reduced the tumor volume, compared to control and low dose celecoxib groups. The rate of lung metastasis was decreased by about 25% in the high dose celecoxib group, compared to the control group. In addition, the area of metastatic lung nodules decreased significantly in the high dose celecoxib group.

With irradiation, COX-2 expression increased in tumors and associated with increased PGE2 levels. Steinauer et al. observed a dose-dependent increase in COX-2, following irradiation. PGE2 levels in irradiated cells were higher than in controls, and decreased when combined with a COX-2 inhibitor [29]. The mechanisms underlying the radiation-enhancing effects of COX-2 inhibitors include (1) an accumulation of cells in the G2/M phases of the cell cycle which are considered to be sensitive to irradiation; (2) a reduction of PG-induced immunosuppressive activity caused by antitumor immunologic responses capable of potentiating tumor responses to radiation; and (3) direct effects on tumor neovascularization [7,30,31]. Shin et al. reported that celecoxib's radiation-enhancing effect was observed in COX-2 expressing cells but was not observed in COX-2 non-expressing cells. The radiation-enhancing effects disappeared in cells treated with COX-2-specific siRNA [32]. LLC cells express COX-2 [26]. In this study, we confirmed COX-2 expression by western blotting. Unlike Steinauer et al., irradiation, regardless of the dosage, did not increase COX-2 expression. However, PGE2 levels increased with irradiation in a dose-dependent manner. Tumor growth was delayed as a result of irradiation, especially in mice treated by irradiation and high dose celecoxib, where tumor growth was markedly delayed. The area of metastatic lung nodules was significantly smaller in mice treated by irradiation, regardless of celecoxib dose, than in the control group. Lung metastases were detected in only 25% of the mice treated by irradiation plus high dose celecoxib, as compared to 100% of the control mice.

The surgical removal of a primary tumor or radiation in order to eradicate a primary tumor can result in rapid growth and metastasis [33,34]. Camphausen et al. reported that a single or hypofractionated irradiation protocol for the eradication of primary LLC cells increased the number of surface lung metastases in irradiated animals, as compared to controls. However, in our study, the number of lung metastases decreased from 100% in control mice to 50% in mice treated with irradiation alone. The differences between these two studies may be due to the doses used, i.e., whether it was a curative dose or not. We did not irradiate the primary tumor for eradication because we were studying the effects of a selective COX-2 inhibitor as a radiation sensitizer for the inhibition of tumor growth and pulmonary metastasis.

In conclusion, a high dose oral treatment of celecoxib significantly inhibited tumor growth, compared to a low dose treatment. In mice treated with radiotherapy and a high dose celecoxib, delay of tumor growth and reduction of pulmonary metastases were more prominent than in the mice treated with celecoxib or radiotherapy alone. However, further studies are needed to evaluate the effect of selective COX-2 inhibitors combined with conventional fractionation or hypofractionation radiotherapy on various cancer cell lines with regard to the delay of tumor growth and inhibition of metastasis.

## Competing interests      

The authors declare that they have no competing interests.  

## Authors' contributions

H.P. participated in the design of the study. J.H.H. carried out pathological analysis in the animal experiment. Y.T.O. participated in its design and coordination and helped to draft the manuscript. W.P. designed the study, performed the experiments and wrote the manuscript.
